# Pilot parallel randomised controlled trial of protective socks against usual care to reduce skin tears in high risk people: ‘STOPCUTS’

**DOI:** 10.1186/s40814-017-0182-3

**Published:** 2017-10-17

**Authors:** Roy J. Powell, Christopher J. Hayward, Caroline L. Snelgrove, Kathleen Polverino, Linda Park, Rohan Chauhan, Philip H. Evans, Rachel Byford, Carolyn Charman, Christopher J. W. Foy, Colin Pritchard, Andrew Kingsley

**Affiliations:** 1 Research and Development Directorate, Noy Scott House, Royal Devon and Exeter NHS Foundation Trust, Barrack Road, Exeter, EX2 5DW United Kingdom; 2 Peninsula Clinical Trials Unit (PenCTU), ITTC Building, Plymouth Science Park, Plymouth, PL6 8BX United Kingdom; 3 University of Exeter Medical School, St Luke’s Campus, Magdalen Road, Exeter, EX1 2LU United Kingdom; 4 NIHR Clinical Research Network, South West Peninsula, Noy Scott House, Royal Devon and Exeter NHS Foundation Trust, Barrack Road, Exeter, EX2 5DW United Kingdom; 5 Research and Development Office, Leadom House, Gloucester Royal Hospital, Gloucester, GL1 3NN United Kingdom; 6 Northern, Eastern and Western Devon Clinical Commissioning Group, County Hall, Topsham Road, Exeter, Devon EX2 4QD United Kingdom; 7 Exeter Clinical Trials Unit (ExeCTU), University of Exeter, RILD Level 3, Barrack Road, Exeter, Devon EX2 5DW United Kingdom; 8 Royal Cornwall Hospital (Treliske), Treliske, Truro United Kingdom

**Keywords:** Skin tears, Pre-tibial lacerations, Prevention, Protective socks, ‘Dermatuff’, Kevlar

## Abstract

**Background:**

Skin tears are common in older adults and those taking steroids and warfarin. They are traumatic, often blunt injuries caused by oblique knocks to the extremities. The epidermis may separate from the dermis or both layers from underlying tissues leaving a skin flap or total loss of tissue, which is painful and prone to infection. ‘Dermatuff™’ knee-length socks containing Kevlar fibres (used in stab-proof vests and motorcyclists’ clothing) aim to prevent skin tears. The acceptability of the socks and the feasibility of a randomised controlled trial (RCT) had not been explored.

**Methods:**

In this pilot parallel group RCT, 90 people at risk of skin-tear injury from Devon care homes and primary care were randomised to receive the socks or treatment as usual (TAU). The pilot aimed to estimate parameters to inform the design of a substantive trial and record professionals’ views and participants’ acceptability of the intervention and of study participation.

**Results:**

Participants were randomised from July 2013 and followed up until February 2015. Community participants were easier to recruit than care homes residents but were 10 years younger on average and more active. To recruit 90 participants, 395 had to be approached overall as 77% were excluded or declined. Seventy-nine participants (88%) completed the trial and 27/44 (61%) wore the socks for 16 weeks. There were 31 skin tear injuries affecting 18 (20%) of the 90 participants. The TAU group received more injuries, more repeated episodes, and larger tears with greater severity. Common daily diary reasons for not wearing the socks included perceived warmth in hot weather or not being available (holiday, in hospital, bed rest). Resource use data were obtainable and indicated that sock wearing gave a reduction in treatment costs whilst well-completed questionnaires showed improvements in secondary outcomes.

**Conclusions:**

This pilot trial has successfully informed the design and conduct of a future definitive cost-effectiveness RCT. It would need to be conducted in primary care with 880 active at-risk, elderly patients (440 per arm). Skin tear incidence and quality of life (from EQ5D5L) over a 4-month period would be the primary and secondary outcomes respectively.

**Trial registration:**

ISRCTN, ISRCTN96565376.

## Background

Skin tears are traumatic wounds involving a piece of skin of varying size being peeled away from underlying tissues either completely or leaving a partial or intact skin flap. They often occur as a result of rubbing, an abrasion or a glancing blow to an arm or leg (e.g. from a fall or being struck or poked obliquely). Until 2011, the most commonly cited definition of a skin tear was that of Payne and Martin: ‘A skin tear is a traumatic injury occurring principally on the extremities of older adults as a result of shearing or friction forces which separate the epidermis from the dermis (partial thickness wound) or which separate both the epidermis and the dermis from underlying structures (full-thickness wound)’ [1]. This was superseded in 2011 [2] by the iSTap (International Skin Tears Advisory Panel) definition ‘A skin tear is a wound caused by shear, friction, and/or blunt force resulting in separation of skin layers. A skin tear can be partial-thickness (separation of the epidermis from the dermis) or full-thickness (separation of both the epidermis and dermis from underlying structures).’ These are common injuries [2–10]; recent data from Japan found that point-prevalence was 3.9% among 410 patients in long-term care [11] and two elderly care rehabilitation units in Australia reported 10% [12]. A non-systematic review [9] reported skin-tear incidence between 2.23 and 41.5% and prevalence between 6.6 and 23.5% in US care homes. In Pennsylvania, skin tear reporting became mandatory for healthcare facilities in 2004 [13], where 88.2% of the 2807 skin tears were in patients aged over 65 years. Fourteen percent of an American 120-bed nursing home population sustained a skin tear per month with an average of 2.67 tears per resident [14]. A recent wound point prevalence audit undertaken in North Devon [15] in 16 care homes revealed 195 wounds among 115 of 458 residents (25%). Traumatic injuries (skin tears) were the second most common wound type (37, 19%) after pressure ulcers (87, 45%).

Rayner et al. [16] conducted a review of the literature to identify studies that described patient and skin characteristics associated with skin tears. The most common patient characteristics from eight published articles and one unpublished study were a history of skin tears, impaired mobility and impaired cognition. Skin characteristics associated with skin tears included senile purpura, ecchymosis and oedema. Several changes occur in the skin that increase its susceptibility to traumatic injury [17, 18]. These changes are due to intrinsic ageing and cumulative extrinsic factors such as photoageing and polypharmacy. They include vascular atrophy and deterioration of the dermis as collagen and elastin fibres become more sparse and disordered, holding the skin layers together less tightly [19]. Older patients may have also taken oral steroids that compromise skin integrity and tensile strength and cause wounds to heal more slowly [20–23]. They may also be less aware that an injury has occurred due to decreased pain perception and tactile sensitivity, including diabetic neuropathy [24]. Fragile skin is most common in people aged over 70. There were 7.4 million people in this age group in the UK in 2011, estimated to increase to 11.2 million in 2030 [25]. Skin tears are unpleasant and provoke anxiety. They can take a long time to heal and are prone to infections. Whilst arm injuries are more common, leg injuries may develop into leg ulcers, which may require lengthy, expensive treatment [26]. Typical causes of skin tears include wheelchairs, other mobility aids, bumping into obstacles, transfers and falls. There are best practice guidelines for treatment and preventing infections and ulcers [27]. Prevention includes staff education, regular assessment, ensuring clothing does not rub, removing obstacles and moisturising the skin [9, 28, 29].

With advancing age, the process of normal wound healing is subject to disruptions and aberrations which can delay the process and predispose the individual to the effects of various factors involved in the formation of a chronic wound [30]. The impact of these changes can be seen in all phases of wound repair, and disruption of any step can lead to an overall delay in healing of between 20 and 60% [31, 32]. Poor management of skin tears can lead to the development of a chronic wound, such as a leg ulcer, with the subsequent impact to the patients’ physical and mental wellbeing [33]. Advancing age, reduced circulation and epidermal turnover rate and also polypharmacy, co-morbidities and nutritional deficits [34, 35] have all been identified as increasing the risk of a chronic wound developing from the initial skin tear.

Skin tears are often treated in the community [36]; however, for more severe tears and if a patient develops complications such as an infection, or is in need of surgical debridement, they will need to be referred to secondary care. In a recent study looking at admissions with skin tears between January 2010 and July 2013 at Charing Cross and Westminster Hospital [36], 73 patients presented with pretibial lacerations, 81% of which were as an acute referral. The remaining patients had previously been treated in the community but the wound either had failed to heal or had developed an infection. Eighty-two percent of the patients presenting required surgical debridement and grafting. Mean length of hospital stay was 11 days for those needing a surgical intervention and 5 days for those undergoing conservative management. These findings are similar to those seen by Rees et al. [37] where the mean hospital stay was found to be 9 days. Three of the patients died before discharge and a further 3 died in the follow up period, with two of the deaths thought to be directly attributable to the skin tears. Another consequence of developing a skin tear is that previously independent living individuals may end up in residential care, up to 20% [37].

One possible way to prevent skin tears may be to wear suitable protective clothing. We describe here a pilot trial of protective socks conceived and developed by a member of the public after receiving many such tears. The protective socks were manufactured by Dermatuff Limited, Woodbury, Devon UK. The socks are CE marked and registered with the Medicines and Healthcare products Regulatory Authority (MHRA) as ‘Skin Tears Protection System Wear’, a class I medical device. Materials used in the manufacture of the socks are in conformity with all relevant and required standards including the ISO 10993 series evaluating biocompatibility of a medical device prior to a clinical study. The socks have a leg section woven from Kevlar [38] and elasticated nylon using the ‘terry sandwich’ method. This gives a flat, slightly ribbed and stretchy, outer woven base which provides a tough, cut and abrasion-resistant exterior. There is a mesh of loops on the inside to provide a cushioning and impact resistant inner layer. The stretchiness is sufficient to fit a range of leg diameters within each size without applying excess pressure, and the socks are held up with a light elastane soft top band. The foot of the socks is manufactured from cotton as laceration protection is not usually required for the feet. Compression hosiery could be worn underneath if required as the socks do not offer any compression themselves. Patients requiring such hosiery were excluded from this study however, as this may confound any protective effects.

Apart from small-scale, uncontrolled testing during development, there has been no trial of the effectiveness and cost-effectiveness of this unique and innovative approach to skin tear prevention.

This pilot study addressed key areas of uncertainty around the running of a full trial by testing the feasibility and acceptability of the research design, methods and proposed outcome measures.

Specific objectives of this pilot study were as follows:

- To determine recruitment, retention and attrition rates
- To consider appropriate outcome measures (including an evaluation of two systems for skin-tear classification), to assess the processes for capturing outcome data and to refine the clinical protocol for a full trial
- To refine the intervention if appropriate through qualitative work on acceptability
- To estimate concordance with the intervention
- To estimate rates of questionnaire completion
- To obtain baseline estimates of scores on the proposed outcome measures in this clinical population and estimate the variability of outcomes to inform the sample size of the definitive trial
- To help establish the eligibility criteria for the future definitive trial
- To estimate the ability to obtain cost and effectiveness measures
- To obtain feedback from care homes regarding the acceptability of the study

## Methods

Detailed methodology including study design, randomisation, eligibility criteria and study protocol are described in a separate protocol paper [39]. The study received approval by the Cornwall and Plymouth NRES Research Ethics Committee.

### Study design

The study was an open, parallel group, pilot randomised controlled study in which participants were randomised in equal proportions to either the ‘intervention group’ (Socks) or the ‘control group’ receiving treatment as usual (TAU). Participants in the intervention group were asked to wear the protective socks during each day (‘waking hours’) for a period of 16 weeks whilst participants in the control group wore their usual clothing. The study was conducted in Devon, UK, and aimed to recruit 90 participants, 45 in each arm. Recruitment was intended to be entirely from care homes, but because of slow recruitment due to low interest in participation and mental capacity problems, an ethics amendment granted in October 2013 allowed further recruitment from the community.

Key outcome measures were collected at baseline, at the end of the 16-week period and within 7 days following any skin tear. Experiences of using the socks and/or taking part in the study were captured through semi-structured interviews with a purposive sample of participants from both the intervention and control arms of the study. Focus groups with care home staff and other professionals captured their perspectives of the study.

Blinding was not possible for participants or research nurses due to the nature of the intervention but was possible for the data analyst by coding the group allocation in the data file.

### Study setting

#### Inclusion criteria

Participants were adults aged 65 years and over at risk of skin tears. Inclusion and exclusion criteria are described in the protocol paper [39].

### Recruitment

Participants were recruited primarily from Care Quality Commission-registered care homes with the advice of the local Tissue Viability Service. Residents of care homes are widely recognised to be an under-researched population and at high risk of suffering skin tears. For logistical reasons, in order to maximise efficiency in term of research nurse resource, recruitment efforts were focused on three geographical areas successively (Exeter, Exmouth/Sidmouth and Mid Devon, roughly representing urban, coastal and rural areas, respectively).

In order to augment recruitment, patients in the community were also invited to participate. Community-dwelling participants were recruited through GP practices supported by the Clinical Research Network (South West Peninsula) or via the ‘Exeter 10000’ research volunteer bank managed by the NIHR Exeter Clinical Research Facility (Exeter CRF). GP staff searched for patients aged 65 years or older who had used oral steroids for more than a month in the prior 12-month period. Lists generated from the search were screened for suitability by a doctor at each participating practice and unsuitable patients excluded, e.g. terminal illness and mental illness. Home visits for consent and for subsequent follow-up visits were sometimes replaced by clinic visits (at a local community hospital or GP surgery for example) if available and mutually convenient.

### Baseline assessment

Research nurses provided potential participants with the approved study information, confirmed eligibility and obtained written informed consent. Subsequently, baseline questionnaires were completed by all participants. These included a standardised measure of health status (EQ-5D-5L) [40], an assessment of capability (ICECAP-O) [41] and an assessment of fear of falling (Short FES-I) [42].

### Randomisation/allocation

Randomisation was achieved by means of a bespoke online system managed by the Peninsula Clinical Trials Unit; participants were allocated to the intervention group or the control group in equal proportions, using blocks of fixed size to generate the allocation sequence and achieve balance in the numbers of participants allocated to each group. Research nurses working in the community were able to access the randomisation service using smart phones.

### Interventions

Three pairs of intervention socks were provided to each participant in the intervention group. The research nurse carried a stock of the socks and provided the correct size in a choice of either charcoal grey or beige colour. Participants allocated to the intervention group were asked to wear the socks during their waking hours every day for a period of 16 weeks, starting from the morning following the day they signed consent, which was designated as ‘day 1’. Care home staff, where applicable, were asked to encourage participants to wear the socks and to assist them to put them on, if necessary. However, it was emphasised that if participants became unwilling to wear the socks or wanted to take them off, they were free to make that choice. Participants continued to wear their normal footwear during participation in the study. Participants allocated to receive routine care were managed as usual. This included any routine procedures to reduce the risk of lacerations, but otherwise they wore their normal clothing.

### Post-randomisation assessments

Participants allocated to wear the socks were given the first of 16 weekly diaries at this visit and were asked by the research nurse to complete the diary on a daily basis. Participants were asked to use the diary to record the extent to which they wore the socks each day and the reasons for not wearing them or removing them (if applicable), plus any negative or positive comments about wearing them. A new weekly diary was provided to the participant each week.

### Feasibility outcome measures

Outcome measures for the pilot study were as follows:

- Recruitment rate for homes
- Proportion of participants (home residents) eligible
- Recruitment rate for participants
- Attrition and loss to follow-up
- Ascertainment of injuries
- Completion and completeness of study questionnaires and diaries
- Estimates of the distribution of outcome measures
- Feasibility of the workload
- Acceptability of the intervention to participants
- Acceptability of study participation to participants

### Outcome measures

The primary outcome measure for a future substantive study was incidence of skin tears (a traumatic injury to the skin of the lower legs resulting in separation of skin layers). Skin tears were reported to the research nurses by care home staff or self-reported by participants in the community. Incidence was also expressed by the number of ‘skin tear-free days’. Each participant entered the study with no unhealed skin tear injuries and remained in the study (unless withdrawn prematurely) for 112 days (i.e. 16 full weeks). Therefore, each participant had the potential to experience 112 skin tear-free days. In the event of a skin tear injury, the number of days from the date of injury until the date it was healed was subtracted from 112 to give the number of skin tear-free days. A healed injury was defined as one which had an absence of scab and full epithelial covering that did not require continuance of dressing for absorption of exudate (sometimes a dressing may be left on a healed but delicate wound for a few days after healing).

Skin tear injuries occurring during the trial were measured using the ‘Visitrak’ (Smith & Nephew, Australia) grid tracing system (length, breadth and area) and classified by trained research nurses using both the Payne and Martin [1] and the Skin Tear Audit Research (STAR) [43] classification systems. Date of healing, description of cause, description of initial management (dressing type, healthcare professional intervention), lower-leg clothing in place at time of injury and, for intervention group participants, the presence or absence of protective socks at the time of injury were all recorded by research nurses for each skin tear injury occurrence. The protocol stipulated that skin tear assessments would be performed within 24 h upon learning of an injury, if possible.

Each injury was photographed by the research nurses according to a trial-specific protocol. Photos were transferred to a blind tissue viability specialist nurse for classification according to the described severity scores. This grading served to inform an assessment of the reliability of the reported grading.

### Secondary outcome measures

The secondary outcome measures were as follows:

- Collected at baseline, 16 weeks and in the event of a skin tear injury:
  - Standardised measure of health status (EQ-5D-5 L)
  - Assessment of capability (ICECAP-O)
  - Assessment of fear of falling (Short FES-I)
- Disease-specific quality of life measured by Cardiff Wound Impact Schedule. Collected in the event of a skin tear injury, within a week after the injury and at 16 weeks (from participants who have had a skin tear)
- Adverse reactions to the socks and serious adverse events
- Skin-tear injury-related healthcare resource use (collected in the event of a skin tear injury)

### Skin tear severity rating scales: inter- and intra-agreement

One of the objectives for the pilot study was to evaluate the two skin tear classification systems available, in terms of their suitability for use in a future substantive trial. All skin tears were therefore assessed and categorised by the research nurses according to both the ‘Payne Martin Classification System for Skin Tears’ [1] and the ‘STAR Skin Tear Classification System’ [43]. Digital photographs were taken of each wound and stored on a secure computer drive within the RD&E Trust. AK made an independent, blind assessment of the categorisation of the wound severity. Intra-rater and inter-rater comparisons were then made.

### Sample size and statistical analyses

Regarding the sample size required for the pilot, in order to estimate a 50% recruitment rate with a 95% confidence interval of +/− 10% points (i.e. an estimate of between 40 and 60% recruitment) within a pool of at least 1000 eligible care home residents for the trial living in care homes in Exeter, Mid and East Devon, a total of 88 patients would be required (44 in each arm). Regarding concordance, if only 50% of recruited patients continue to use the socks for the duration of the trial, 90 patients would enable us to estimate that with +/− 10% precision (from 40 to 60% concordance). StatsDirect 2.6.6 (Altrincham, UK), which used the methods of Colton [44] and Feinstein [45].

We report and present data according to the relevant CONSORT statement [46]. The primary analyses were all pre-specified in a detailed statistical analysis plan approved by Trial Steering Group before the analyses started. As this was a pilot study, a purely descriptive analysis was undertaken. The study data were analysed using the statistical package SPSS v. 23 (IBM Corp, New York). Missing data were investigated and the proportions missing were recorded. Multiple imputation methods or specific imputation methods recommended by the authors of the questionnaires were used to obtain realistic estimates of scores for future planning of questionnaire usefulness. All the questionnaires concerning acceptability of the socks were scored and summarised using appropriate measures of central tendency and dispersion. Data on lacerations were also summarised in a similar way. Agreement on wound grading between research nurses and a blinded tissue viability expert (AK) was assessed from anonymised photographs using Cohen’s weighted kappa. Numbers of eligible residents, recruitment, attrition and loss to follow-up (as per the Consolidated Standards of Reporting Trials (CONSORT) diagram) were reported as proportions and confidence intervals wherever appropriate. The main analysis described skin tear-free days and the incidence and severity of skin lacerations in each group.

The pilot study was intended to determine the feasibility of how the outcomes can be measured. These were used to inform the sample size of the future substantive randomised controlled trial. In that trial, we intend to compare the incidence of skin damage between the two groups on an intention-to-treat basis and the size of any wounds by objective assessors (research nurses).

### Patient involvement

The trial intervention socks were conceived and developed by a member of the public who has suffered from skin tears himself. During the design of this study, a separate patient representative contributed to the development of the grant application and, later, to the study protocol and participant facing documentation after funding had been awarded. There were also other patient representatives on both the Trial Management group and on the Trial Steering Committee, who helped to oversee progress of the trial and provided a patient’s perspective on aspects of trial conduct. A lay summary of the study findings will be made available to participants at www.medicalresearchplymouth.org.uk.

### Adverse and serious adverse events

Any adverse events which were serious and/or related to study procedures or the socks were recorded. Multiple symptoms were recorded as separate events. The events were reported to the Chief Investigator, Sponsor and the Peninsula Clinical Trials Unit on a designated report form which captured the research nurse’s opinion on the relatedness of the event to study procedures/intervention and also on the expectedness of the event. Adverse reactions to the socks were expected to be uncommon. The following list of potential symptoms was used as a reference when assessing the expectedness of adverse device effects.

- Allergic-type skin reaction
- Miliaria (heat rash)
- Chafing
- Excessive sweating under the socks
- Skin tears or bruising caused by putting on or removing the socks
- Pain, discomfort, numbness, swelling or any other condition caused by socks which were too tight
- Falls or other accidents caused by slipping or tripping as a result of wearing the socks

Cumulative summaries of adverse reactions were reviewed periodically by the Trial Steering Committee.

### Economic evaluation

The economic evaluation in the future substantive trial will estimate the additional NHS cost per QALY (quality-adjusted life years) gained by the use of these protective socks. In this pilot, QALY estimations were based on the EuroQol descriptive system (EQ-5D-5L) collected at baseline and at 16 weeks for all participants and at 7 days after a participant incurred a skin tear injury to the leg.

The study assessed the ability to obtain cost and effectiveness measures. It aimed to collect data on the resources used by the care homes, visits to/from GPs or healthcare professionals and the NHS in the management of skin tears. The primary source of these data were the participants’ medical records of their normal procedures, visits from district or tissue viability nurses, visits to and from GPs and any care needs arising from adverse events. The pilot study assessed whether care home records and or participants’ medical notes were adequate to describe resource use in a costable format.

### Qualitative interviews and focus groups

Semi-structured qualitative interviews with 20 participants from both study arms were conducted at the end of the follow-up period by an independent researcher. These included a range of participants with varying degrees of mobility across the geographical areas. Experiences of using the protective socks, their acceptability and/or taking part in the study were captured. Participants were selected using purposive sampling informed by research nurse data on their clinical progress with regard to skin tears, perceived protection from knocks and falls, withdrawals, adverse events and any problems with the skin-tear and/or questionnaire data collection process. In order to get a representative sample, a range of people across different ages and gender were interviewed as per the inclusion criteria. Interviews were digitally recorded and transcribed with the main themes identified using content analysis.

Two focus groups were convened to explore the usefulness of the protective socks. These groups included professionals from the Tissue Viability Service and care home staff with experience of participants assigned to the intervention arm of the study. Holding two focus groups provided easy access for staff of care homes across the large geographical area of the pilot trial.

## Results

The first participant was randomised on 30th July 2013 and the last on 2nd October 2014. Follow-up was completed in early February 2015. A total of 90 participants were recruited, with 44 randomised to the intervention arm and 46 to the TAU group.

### Baseline characteristics

Table 1 provides baseline characteristics of participants and demonstrates reasonable balance between the two randomised arms, although a slightly higher proportion of women were allocated to the intervention group.

**Table 1:** Baseline characteristics

	Protective socks (*n* = 44)	Treatment as usual (*n* = 46)	All (*n* = 90)
Demographics			
Female sex	24 (54.55%)	18 (39.13%)	42 (46.7%)
Age, years, median (IQR)	86 (12)	85 (11)	85.5 (11)
Care home residents	27	27	54 (60%)
Female sex	14 (51.85%)	12 (44.44%)	26 (48.15%)
Age, years, median (IQR)	90 (8)	88 (9)	90 (9)
Community participants	17	19	36 (40%)
Female sex	10 (58.82%)	6 (31.58%)	16 (44.44%)
Age, years, median (IQR)	79 (16)	80 (11)	80 (12)

### Recruitment and eligibility

**Fig. 1:**
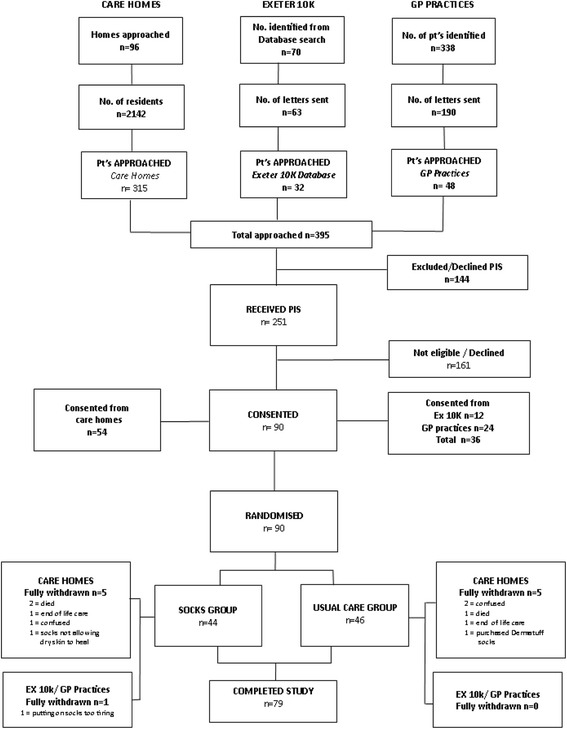
CONSORT Diagram

**Fig. 2:**
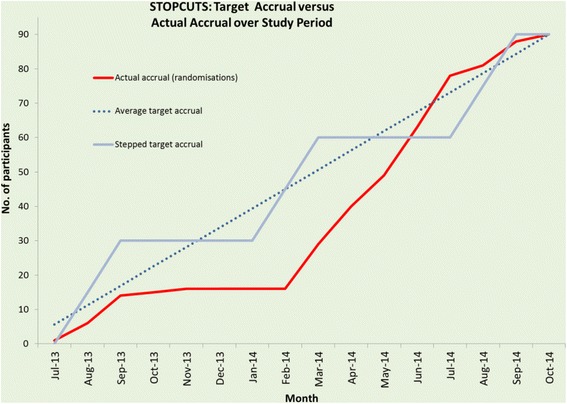
Target vs actual recruitment

See CONSORT diagram (Fig. 1) and recruitment graph (Fig. 2). Ninety-six care homes were identified from the CQC database and were approached. They contained 2142 residents, of which 315 suitable residents (15%) were identified by care home managers and approached by the research nurses. In the community, 338 patients were identified from GP databases and screened. Of these, 190 (56%) were sent letters about the study on Surgery headed paper and 48 of these (25%) were approached. A further 70 volunteers were identified from the Exeter 10 K volunteer database. Of these, 63 (90%) were sent letters about the study and 32 of these (51%) were approached.

Of the 395 people approached, 144 (36%) were excluded or declined to take part and 251 participants (64%; 95% CI 59 to 68%)) received a Participant Information Sheet (PIS). Ninety of these (36%; 95% CI 30 to 42%) gave informed consent (54 in care homes (60%), 24 from GP practices (27%) and 12 volunteers from Exeter 10 K database (13%)). Of the 54 in care homes, 16 were from the Exeter area and 38 from Sidmouth/Exmouth.

Only 54 (60%) of the participants could be recruited from care homes because of residents’ reluctance to take part in research or mental capacity issues. The remaining 36 (40%) were recruited from the community after ethics amendments (see Fig. 2). Furthermore, 96 CRC approved care homes and 315 residents had to be approached to give a recruitment rate in care homes of 17%, 95% CI 13 to 22%. Among the three sources of participants, the highest conversion from ‘approached’ to ‘recruited’ was achieved in GP practices (50%; 95% CI 35 to 65%). The lowest conversion was in care homes (17%; 95% CI 13 to 22%). In volunteers, it was 37.5%, 95% CI 21.1 to 56.3% (*P* < 0.0001). In summary, in order to recruit 90 participants, 395 had to be identified and approached by the research nurses (approx. 1 in 4).

### Socks

Sixteen women (67%) requested (and were fitted with) beige socks and 8 (33%) women with grey socks. None of the women required the large size. Thirteen (63%) of the men requested (and were fitted with) beige socks and 7 (37%) with grey. Most of the men (63%) required the large size, and none of them had the small size. The most common size used in this trial was standard-medium (37% of participants) and colour beige (65%).

### Concordance, retention and attrition

Of the 44 participants in the socks group:

- 27 participants (61.4%) wore the socks for the full 112 days.
- 17 participants (38.6%) discontinued wearing the socks.
- 6 participants (13.6%) withdrew from the trial altogether.

In the control group:

- 41 participants (89.1%) completed the trial (112 days).
- 5 participants (10.9%) withdrew.

The results are shown in Table 2. Of the 17 participants who stopped wearing the socks, this occurred a median of 14 days from allocation (IQR 27 days). Two individuals stopped wearing them on the first day (day 0) because they did not fit properly: the foot part was too large or socks were too long.

**Table 2:** Compliance with wearing the Dermatuff™ socks

Reason	Stopped wearing socks	Withdrawn
Socks uncomfortable	7	
•1 legs swell up		
•2 socks too long		
•2 socks too warm (1 same person as for socks too long),		
•1 socks too tight		
•1 developed blister		
•1 socks itched		
Participant too unwell (1 dying, 1 chronic illness and too tired to put on socks)	2	2
Participant lost capacity	2	2
Previous skin problem (dry skin on heels)	1	1
Found socks unsightly/embarrassing	1	
Socks did not fit (thin calves)	1	
Died	1	1
No reason given	2	
Total	17	6

### Primary clinical outcome: skin tear injuries

A list of all the skin tears is given in Table 3, with summary data reported in Table 4. During the course of the trial, there were 31 skin tear injuries affecting 18 of the 90 participants in the trial (20%) over a period of 112 days—8 people out of 44 (18.2%; 95% CI 8.2 to 32.7%) in the socks group and 10/46 (21.7%; 95% CI 10.9 to 36.3%) in the control group. Seven of the 18 first-time tears (38.9%) occurred within the first month. Six participants had repeated skin tears, and the maximum number of repeated tears was 8. The outcome measure ‘skin tear-free days’ was highly skewed because 80% of participants in the trial experienced no injuries (112 skin tear-free days). The median (IQR) time to the first injury was 38 (29) days in the socks group and 28 (63) days in the TAU group. The hazard ratio (95% CI) of group 1 (socks) vs. group 2 (TAU) = 0.81 (0.32 to 2.04).

**Table 3:** Reported skin tear injuries

Participant number	Age	Sex	Tear	Allocation	Care home resident	Date randomised	Time to injury (days)	Date of injury	Time of injury	Cause of injury	Wound surface area (cm2)	STAR grading	PAYNE-MARTIN grading	Wearing on legs at time of injury	Time taken to heal (days)
16	87	M	1	Socks	Y	14/11/13	8	22/11/13	20:50:00	Fall	1.1	1a	Ia	Nothing	17
32	91	M	1	Socks	Y	08/04/14	95	12/07/14	19:20:00	On Zimmer frame transferring from chair to bed	1.4	1b	Ia	Trousers, non-intervention Socks	10
*41	99	F	1	Socks	Y	01/05/14	45	15/06/14	15:00:00	Unknown cause. Could not remember injury	3.6	2b	IIb	Not known	26
64	87	F	1	Socks	Y	02/07/14	39	10/08/14	01:00:00	Fell out of bed	3.1	2b	IIa	Non-intervention socks	32
62	68	M	1	Socks	N	17/06/14	12	29/06/14	01:00:00	Knocked R leg on door frame	0.31	1a	Ia	Nothing	8
65	73	F	1	Socks	N	03/07/14	32	04/08/14	Unknown	Overnight	2	2b	IIa	Nothing	24
79	86	M	1	Socks	N	04/08/14	46	19/09/14	Unknown	Penetrating wound	0.2	NK	NK	NK	63
79	86	M	2	Socks	N	04/08/14	72	15/10/14	15:00:00	Dropped a heavy loaded crate against leg	0.31	3	III	Trousers	36
*85	81	F	1	Socks	N	17/09/14	39	26/10/14	13:00:00	Brambles in garden dragged against leg	4	1a	Ib	Intervention socks	31
*85	81	F	2	Socks	N	17/09/14	43	30/10/14	09:00:00	Knocked L shin against plant pot in garden	1.6	2b	IIb	Trousers and intervention socks	27
29	92	M	1	Usual care	Y	31/03/14	58	28/05/14	Unknown	Unknown cause. Could not remember injury	0.55	3	III	Not known	12
30	98	M	1	Usual care	Y	03/04/14	25	28/04/14	Unknown	Knock to leg	2	1a	Ib	Trousers	17
46	86	M	1	Usual care	Y	08/05/14	1	09/05/14	18:30:00	Fall on transfer from chair to bed	0.8	1b	Ib	Trousers	18
46	86	M	2	Usual care	Y	08/05/14	87	03/08/14	11:30:00	Fall	6.1	2a	IIa	Trousers	23
47	94	F	1	Usual care	Y	12/05/14	1	13/05/14	11:45:00	Unknown cause. Could not remember injury	6.4	2b	IIb	Trousers	27
47	94	F	2	Usual care	Y	12/05/14	107	27/08/14	15:30:00	Whilst using bathroom	2.2	1b	Ia	Non-intervention socks	13
47	94	F	3	Usual care	Y	12/05/14	108	28/08/14	10:00:00	Unknown cause. Could not remember injury	1.2	2b	IIa	Non-intervention socks	12
75	86	F	1	Usual care	Y	24/07/14	12	05/08/14	15:00:00	Knocked leg on edge of sharp object. Refused tracing.	Unknown	1b	Ib	Nothing	180
55	73	F	1	Usual care	N	10/06/14	83	01/09/14	08:30:00	Fell on slippery surface	0.32	3	III	Trousers	15
55	73	F	2	Usual care	N	10/06/14	106	24/09/14	14:00:00	Dropped a cardboard box against leg	0.4	2b	Ib	Trousers	36
57	88	M	1	Usual care	N	12/06/14	100	20/09/14	14:00:00	Caught R shin on edge of a door	0.5	3	III	Shorts	20
59	82	M	1	Usual care	N	17/06/14	31	18/07/14	Unknown	Knocked leg in garden	0.5	2a	III	Trousers	10
70	83	M	1	Usual care	N	14/07/14	6	20/07/14	Unknown	Fingernails on pulling up socks	2.2	2a	IIa	Nothing	24
70	83	M	2	Usual care	N	14/07/14	32	15/08/14	11:35:00	On wine rack whilst walking past	2	2a	IIa	Nothing	11
70	83	M	3	Usual care	N	14/07/14	46	29/08/14	09:30:00	Brushed against bed frame	2.8	2b	IIa	Nothing	17
70	83	M	4	Usual care	N	14/07/14	46	29/08/14	15:30:00	Whilst mowing lawn	2.6	2b	IIa	Non-intervention socks	17
70	83	M	5	Usual care	N	14/07/14	54	06/09/14	15:00:00	Knocked leg whilst gardening	1.9	2b	IIa	Trousers	13
70	83	M	6	Usual care	N	14/07/14	59	11/09/14	02:30:00	In bed pulling sheets over self	2.75	3	III	Nothing	20
70	83	M	7	Usual care	N	14/07/14	65	17/09/14	15:00:00	Fell in garden	15.5	2b	IIa	Shorts	29
70	83	M	8	Usual care	N	14/07/14	65	17/09/14	15:00:00	Fell in garden	33.1	3	III	Shorts	29
89	71	F	1	Usual care	N	01/10/14	63	03/12/14	11:00:00	Knocked leg on edge of recycling bin	1.3	1a	Ib	Trousers	14

*Participant was definitely wearing intervention socks at the time of injury

**Table 4:** Reported skin tear injuries: incidence, skin tear-free days (STFD), size and severity using Payne-Martin (PM) and STAR grading systems

	Socks group	TAU group	Total
Total allocated	44	46	90
Participants with skin tears	8 (18.18%)*	10 (21.74%)	18 (20%)
Total number of skin tear injuries	10	21	31
No. with 1 injury	6	6	12
No. with 2 injuries	2	2	4
No. with 3 injuries	0	1	1
No. with 8 injuries	0	1	1
Incidence (people suffering tears)	8/44 = 0.18	10/46 = 0.22	RR = 0.84 (0.37 to 1.88)
Incidence (tears/episodes of care)	10/44	21/46	RR = 0.50 (0.26 to 0.91)
Mean (sd) total area of injury (cm^2^)	1.76 (1.39)	4.26 (7.61) (missing data on case 3001)	
Median (IQR) total area of injury (cm^2^)	1.5 (2.92)	2 (2.18) (missing data on case 3001)	
Median (IQR) duration (days)	26.5 (17.75)	17 (10.75) [missing data on case 3001]	
Mean (sd) STFD for whole group (days)	107.2 (12.43)	105.72 (16.5)	
Median (IQR) STFD for whole group (days)	112 (0)	112 (0)	
PM category Ia	3	1	4
PM category Ib	1	4	5
PM category IIa	2	8	10
PM category IIb	2	2	4
PM category III	1	6	7
PM category missing	1		1
STAR category 1a	3	2	5
STAR category 1b	1	3	4
STAR category 2a		4	4
STAR category 2b	4	7	11
STAR category 3	1	5	6
STAR category missing	1		1

*Participant was definitely wearing intervention socks at the time of injury

The usual care group received more tear injuries, more repeated episodes, larger tears (socks group, median 1.5 cm^2^ (IQR 2.92 cm^2^); TAU median 2 cm^2^ (IQR 2.18 cm^2^)) and more severe tears which would require emergency treatment (STAR grade 3: socks group 1/10 (10%); TAU group 5/21 (24%)). If STAR grade 2b and 3 are combined, this amounts to socks group 5/10 (50%); TAU 12/21 (57%).

Among the 54 care home residents, nine (17%) received tears: four in the socks group (one tear each) and five in the TAU group (total eight tears). In the community, nine of the 36 participants (25%) suffered a tear: four in the socks group (total six tears) and five in the TAU group (total 13 tears).

Only 2 of the 8 people with skin tears in the socks group (cases #41 and #85, marked with * in Table 3) were definitely wearing the intervention socks at the time of the injury. Also, one of these (#41) was noted by care home staff to habitually roll their socks down. Their injury occurred during the hot summer of 2014 when UK temperatures reached 27 °C [47].

Causes of the skin tears differed between the care home and community participants. In care homes, the 12 tears were caused by falls (4) including falling out of bed and in transferring from bed to chair; knocks against objects (4) such as walking aids, bathroom furniture and blunt or sharp edges and ‘unknown’ (4) where the participants could not recall the cause. In the community, the 19 tears were caused by gardening activities (7) including two falls in the garden; knocks against objects (4) such as a door, a door frame, wine rack and recycling bin; overnight in bed (3) on the bed frame and simply by dragging bed sheets over their legs; dropping objects being carried (2)—a full cardboard box and a heavy crate that scraped the legs; and ‘other’ (3) such as putting ordinary socks on in the morning (from fingernails), a penetrating injury of unknown origin and a fall indoors.

### Skin tear severity rating scales: inter- and intra-rater agreement

Table 5 gives reliability data for the two severity rating scales. There was good agreement between the Payne Martin and STAR classification systems, whether assessed by a nurse (kappa = 0.7) or a tissue viability expert (kappa 0.65). However, the inter-rater reliability, comparing nurse with expert, was only ‘fair’, for both Payne Martin (kappa 0.26) and STAR (kappa 0.22).

**Table 5:** Reliability of Payne-Martin and STAR grading scales for skin tears

	Inter-rater agreement: nurses vs. tissue viability expert (AK)		Inter-scale agreement: Payne-Martin vs STAR	
	Payne-Martin Groups 1a, 1b, 2a, 2b and 3	STAR Groups 1a, 1b, 2a, 2b and 3	Nurse grading	Tissue viability expert (AK) grading
Observed agreement	75%	74.1%	88.33%	90%
Expected agreement	66.2%	66.8%	61.67%	71.39%
Kappa +/− 95% CI	0.26 (0.02 to 0.5)	0.22 (− 0.02 to 0.46)	0.70 (0.55 to 0.84)	0.65 (0.47 to 0.84)
*p*	0.0118	0.0264	< 0.0001	< 0.0001

### Adverse events

Non-serious adverse events were reported only if a possible causal relationship to intervention or trial participation was suspected. No events were attributed to trial participation. Twelve events were reported as having a causal relationship to the protective socks: 8 were classified as being mild and 4 were classified as moderate in severity (Table 6).

**Table 6:** Non serious adverse events listed in order of increasing severity

Summary	Severity	Related to socks?	Resolution
Rubbing and red marking on tips of big toes	Mild	Possibly	Recovered
Itching and discomfort of lower legs and feet	Mild	Probably	Recovered
Unconfirmed fungal infection of heel	Mild	Possibly	Recovered
Dry, excoriated skin	Mild	Possibly	Recovered
Lower leg discomfort	Mild	Definitely	Recovered
Lower leg discomfort	Mild	Definitely	Recovered
Itchy leg, particularly at top of sock area	Mild	Probably	Recovered
Worsening of pre-existing rash	Mild	Possibly	N/K
Pain, discomfort and swelling	Moderate	Probably	Recovered
Toe and foot discomfort	Moderate	Possibly	Recovered
Itchy legs, blister on 3rd toe left foot	Moderate	Probably	Recovered
Pain in left shoulder	Moderate	Possibly	Recovered

As a safety measure, all serious adverse events were reported regardless of relatedness to trial participation or intervention. Ten serious adverse events (SAE’s) were reported, none of which were regarded as being a reaction to the socks or caused by taking part in the trial.

### Completeness of diary data

Of the 704 weekly diaries given out, 482 (68.5%) were returned. All diary comments were documented, reviewed and categorised to determine how the information would be reported. Most of the unobtainable diaries were due to participant withdrawals. The number of diary days that had comments written on them varied between 0 (4 participants) and 99 (1 participant). Six hundred eighty comments were recorded in total.

The diaries encompassed a total of 4928 participant days for those allocated to the socks (2688 for women and 2240 for men) to write comments. Women wrote on 417 participant days (15.51%) and men on 263 participant days (11.74%). Only 6 participants wrote comments on 30 or more days of the 112-day follow-up period. Themes from these comments were coded and these are summarised in Table 7. The commonest comment concerned the perceived warmth of the socks (women 190 comments (45.6%); men 112 comments (42.6%)) and in particular that the weather was too warm to wear the socks. However, to put this into context, these represent 7.1% of the total participant days for women and 5% for men. Ninety-nine such comments came from one participant.

**Table 7:** Diary comments summarised and frequencies provided by men and women

Comment	Women			Men		
Reasons for not wearing socks and other comments (summarised)	Frequency	Percent	Valid percent	Frequency	Percent	Valid percent
All pairs in the wash	14	0.52	3.36	4	0.18	1.52
Bed rest: not wearing socks	5	0.19	1.2	16	0.72	6.08
Chiropodist appointment	3	0.11	0.72	5	0.22	1.9
Diary day not completed	2	0.07	0.48	1	0.04	0.38
Difficult to put on				2	0.09	0.76
Feet and legs creamed	6	0.22	1.44	4	0.18	1.52
Foot swollen/shoe fitting issue	5	0.19	1.2	4	0.18	1.52
Forgot to wear the socks	7	0.26	1.68	2	0.09	0.76
Legs tend to swell	2	0.07	0.48			
In hospital	1	0.04	0.24	9	0.4	3.42
Itching	1	0.04	0.24	6	0.27	2.28
Making legs swell/ft sweaty	1	0.04	0.24			
Not wearing socks—support hose	2	0.07	0.48			
Not wearing socks—trousers	1	0.04	0.24			
Not wearing socks—went out	19	0.71	4.56			
Not wearing socks—skirt	5	0.19	1.2			
Not wearing socks—other	10	0.37	2.4	2	0.09	0.76
On holiday	32	1.19	7.67	10	0.45	3.8
Ongoing fungal infection				3	0.13	1.14
Out all day	9	0.33	2.16	1	0.04	0.38
Physio appointment				5	0.22	1.9
Podiatry appointment				1	0.04	0.38
Positive: do not rub toes raw unlike conventional socks				2	0.09	0.76
Positive: felt socks prevented injury from a knock	2	0.07	0.48	2	0.09	0.76
Positive: very comfortable	5	0.18	1.2	2	0.09	0.76
Positive: socks ease my leg swelling	1	0.04	0.24			
Refused	11	0.41	2.64			
Skin tear on leg	4	0.15	0.96			
Too hot	81	3.01	19.42	25	1.12	9.51
Too hot: took the socks off	14	0.52	3.36	10	0.45	3.8
Too hot: weather too warm	95	3.53	22.78	77	3.44	29.28
Took the socks off for wound check	6	0.22	1.44	2	0.09	0.76
Uncomfortable/ill-fitting	6	0.22	1.44	3	0.13	1.14
Unhealed lesion				1	0.04	0.38
Unwell	3	0.11	0.72	2	0.09	0.76
Wearing sock on one leg only	13	0.48	3.12	22	0.98	8.37
Wearing socks as weather cool enough	4	0.15	0.96	2	0.09	0.76
Wearing socks on both legs	5	0.19	1.2	2	0.09	0.76
Wore socks in the evening only	4	0.15	0.96	1	0.04	0.38
Wore socks some of the day	22	0.82	5.28	24	1.07	9.13
Wore the socks all day	16	0.6	3.84	8	0.36	3.04
Other				3	0.13	1.14
No comment recorded	2271	84.49	Missing	1977	88.26	Missing
Total	2688	100	100	2240	100	100

A range of other comments were expressed including wearing the socks only part of the day, wearing only one sock (presumably after a treatment to one leg) and not wearing them because the participant was either on holiday, in hospital or taking bed rest. Positive comments related to comfort and possibly offering protection from some knocks.

### Economic evaluation

Whilst the number of different types of health professionals seen and the number of occasions were recorded, it was not possible to obtain precise timings and durations of treatments provided by them as clinical records were not easily obtainable in the time available. However, the costs of visits to and from healthcare professionals were calculated using Personal Social Services Research Unit estimates of costs [48]. EQ5D5L data and derived variables where appropriate were non-normal (positively skewed), so estimates of the distributional characteristics were obtained using repeated sampling (bootstrap) methods.

The use of the protective socks resulted in reductions in the incidence of skin tears. The trial incidence of skin tears was 0.2029 per 100 person days in the group allocated to wear the socks and 0.4076 per 100 person days in the usual care group (odds ratio for sock wearing 0.2250 95% CI 0.0881 to 0.5748). The bootstrap median estimate of injuries averted per 100 person days in the sock wearing group was 2 (inter-quartile range 1 to 4).

#### Severity of injuries

Although the severity scores [1] do not differ between the groups, sock wearing may be associated with a reduction in the length of time taken for skin tears to resolve (average 30.33 (s.d. 30.45) days for the injuries in the sock wearing group and average 37.20 (s.d. 45.10) for injuries in the usual care group).

i. Quality of life

Over the trial period, wearing socks was associated with a higher health-related quality of life. Quality of life was assessed using EQ-5D-5L [40] and the OHE tariff [48]. The baseline quality of life was similar and quite low for STOPCUTS participants (mean baseline tariff value 0.66 (s.d. 0.23) for the sock wearers and 0.67 (s.d. 0.26) for the usual care group). The mean within trial QALY—with ‘perfect health’ over 6 months giving a maximum value of 0.5 QALY—was 0.211 (s.d. 0.076) for sock wearers and 0.206 (s.d. 0.067) for the usual care group. The bootstrap estimate of the median QALY deficit associated with usual care was 0.065 (inter-quartile range 0.028 to 0.10).

ii. Costs

**Fig. 3:**
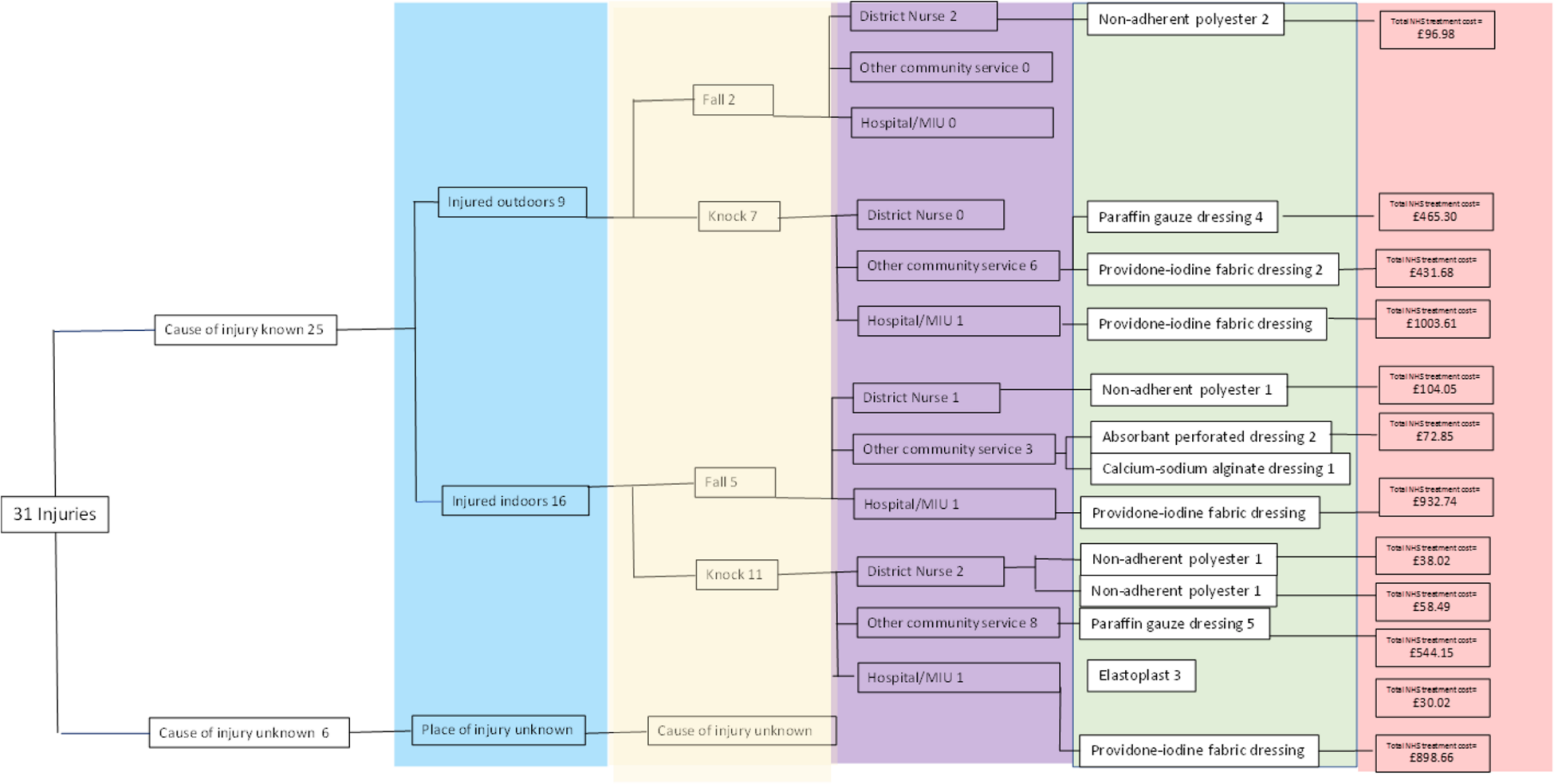
Decision tree for all injuries: occurrence, where and how treated by whom, with associated costs

Wearing the protective socks resulted in the group incurring lower NHS costs for the treatment of skin tears. The resources used to treat skin tears occurring during the trial are shown in Fig. 3. The costs shown in the diagram are estimates of the total NHS costs for treating all the injuries in that branch of the decision tree until they were resolved. The costs include the costs of the health professionals’ time and the NHS prices for the dressings used. Both of these costs depend on the time taken for injuries to resolve—the duration of treatment. The time taken for the injury to resolve varied for the injuries recorded in the STOPCUTS study. The shortest time for an injury to resolve was 8 days (Payne Martin Grading of the injury was 1a) and the longest 180 days (Payne Martin grading of the injury was 1b). The average time taken for injuries to resolve was 26.81 days (standard deviation 30.48). Overall, district nurses opted for similar dressings to nurses from other specialisms. The main NHS resources used were, firstly, the specialised dressings used. Following guidelines [49], the dressings used depended on the amount of skin lost in the injury, the quantity of exudate and the general condition of the patient’s skin. The dressings used were non-adherent and ranged from transparent film dressings to silicone-based foam dressings, costed at prices to the NHS. Reflecting the severity and duration of the injury, the dressing costs per injury were £37.28 for injuries in the sock wearers and £82.35 for injuries in the usual care group. For all trial participants, including those who were not injured, the dressing costs were £7.63 for the sock wearing group and £15.71 for the usual care group.

The second major component of the NHS costs was the NHS staff time needed to treat the injury. There were 107 visits to or from healthcare professionals by the 18 people (31 skin tears) who were injured during the trial period. Most of the visits were from community-based nursing staff (District nurses or tissue viability specialist nurses). There were three visits to minor injury units and two emergency department attendances. There were 54 general practice attendances and patients were seen by their General Practitioner on 21 occasions. In STOPCUTS, none of the injuries required a hospital admission or plastic surgery treatment although both of these higher cost treatments are sometimes needed for treating skin tears.

The average cost of healthcare staff attendances was £153.44 (sd £293.19) per injury for the sock wearing group and £234.60 (sd £403.18) per injury for the usual care group. The cost distributions were heavily positively skewed: the median costs per injury were much lower at £13.00 (inter quartile range (IQR) £13.00–£174.00) for injuries in the sock wearers and £19.50 (IQR £9.75–£372.25) for injuries in the usual care group.

The estimate of total NHS costs included two pairs of the protective socks costed at £30.00 per pair. On this basis, average costs per person for all sock wearing participants and injury treatment were £60 + £39.01 respectively, totalling £99.01 (sd £157.49) and £66.38 (sd £257.74) per person for all usual care participants. Reflecting the fact that the majority of participants in both groups were not injured, median costs were £60.00 for the sock wearers and £0.00 for the usual care group.

What the STOPCUTS study revealed about the actual treatment of skin tears in the community was that:

- Skin tear injuries happened in several different ways in a variety of settings.
- The injuries were not always immediately recognised by the patients or their carers.
- The initial treatment of the wound was undertaken by health professionals from a range of specialisms.
- Overall, most of the care was provided by district nurses visiting their patients in their usual place of residence.
- Although there was a relatively limited set of dressing technologies used, an assortment of different brands of dressing were used.
- There was substantial variation in the time it took for the wounds to resolve—the duration of treatment; 16.1% of the injuries took more than a month to heal.
- There was no clear-cut relationship between the initial assessment of the severity of the injury as assessed by STAR or Payne-Martin classifications and the duration of treatment.
- The heterogeneity of cost-sensitive aspects treatment regimes was reflected in the spread distribution of the estimates of the NHS costs incurred by a skin-tear injury—these are the estimates of the costs that were averted when the injury was prevented by the use of the thin skin protection socks.

### Interviews and focus groups

Twenty participants from care homes and primary care were interviewed (13 intervention and 7 TAU group). Participants in the intervention group had been asked about their experiences of wearing the socks, and members of both groups were asked how they felt about taking part in the trial. Saturation was reached in the TAU group after 7 interviews. Three main themes arose from participant interviews: theme 1, ‘General Involvement in the study’—impact on quality of life. The sub-themes were (a) ‘Impact on everyday life’—social life family and friends; (b) ‘Motivation to take part’ and (c) ‘Coping with being involved’. Some participants reported having enjoyed taking part in the trial and that health professionals were impressed that they had participated. Motivation to take part was often influenced by knowing people who suffered from skin tears and leg ulcers. In some interviews, people mentioned having felt a sense of altruism that had motivated them to take part in research that might help sufferers even if it did not benefit them directly. Others felt a little bothered about filling in the diary about the sock wearing.

Theme 2 was the trial-specific experience related to communication and support. The subthemes were (a) Information given and (b) experience of being involved relating to support and other issues. Participants remembered reading the information, feeling fully informed about the trial and giving their consent. They felt that the support from the research nurses was very good—especially the regular contact with them. This included those who had to drop out because they could not wear the socks. People were happy to be randomised on the whole.

Theme 3 was the experience of wearing the socks with subthemes of (a) general feedback, (b) specific issues and (c) quality of life. Putting on and taking off the socks without help from carers was an issue for some participants, e.g. one had a shoulder problem, preventing him from putting them on. However, another care home resident bought himself a simple device to aid in putting on socks. Once the socks were on, wearers mostly found them comfortable. Some participants would not consider wearing them to socialise with smart clothing. Several people made comments about the perceived warmth of the socks, saying that they would be happy to wear them in winter but not in the summer (bearing in mind that the summer of June 2014 was the hottest on record). Certain participants felt the foot of the sock would be loose and ‘baggy’ so that putting shoes on was difficult. It was felt that some refinement of the design might be beneficial. People generally felt protected by the socks, however. Participants commented on the benefits of wearing the socks. In some cases, they were aware that they had knocked themselves but had no marks or bruises on their legs when they took the socks off.

In the focus groups with care home staff and professionals, care home staff were pleased that their homes had had the opportunity to take part in a clinical trial. They commented on how well it was conducted and how efficient and unobtrusive the research nurses were. Some staff asked why residents of homes with dementia were excluded from the trial. They felt that these were more prone to suffering from skin tears because of their reduced awareness of the dangers and physical obstacles. Other comments were made about the potential benefits of residents wearing the socks in bed because they injure themselves at night too, perhaps just as leggings rather than a complete sock.

### Secondary outcomes: questionnaires at baseline, after injury and at completion

Table 8 gives data on secondary outcome measures captured through questionnaires. Of the questionnaire booklets, 90 (100%) were completed at baseline and 88% at 16 weeks. There were 11 (12%) unobtainable questionnaire booklets at 16 weeks (and none overdue), providing full data on 79 (88%) participants (38 socks group; 41 TAU). The best completed measure as a whole was the EQ5D5L with 100% returned both at baseline and at 16 weeks. The least favoured measure was the ICECAP-O, especially for care home residents because it forced them to focus on the negative things in their lives and raised issues which they found unpleasant.

**Table 8:** Secondary outcome measures

	Socks group: percent completion, mean and (s.d.) or median and (IQR). *N* = 44				TAU group: percent completion, mean and (s.d.) or median and (IQR). *N* = 46			
Measure Completion (%) Mean (sd) Median (IQR)	Baseline	After injury (*n* = 10)	At 16 weeks	Change from baseline to 16 weeks	Baseline	After injury (*n* = 13)	At 16 weeks	Change from baseline to 16 weeks
EQ5D mobility	100%	100%	86.4%		100%	61.9%	89.1%	
	3.09 1.25)	3.10 (1.37	2.74 (1.31)	− 0.35	2.83 (1.36)	2.38 (1.12)	2.56 (1.31)	− 0.27
	3.5 (2)	3.5 (2)	3.0 (3)	− 0.5	3.0 (2)	2.0 (2)	3.0 (3)	0
EQ5D self-care	100%	100%	84.1%		100%	61.9%	87%	
	2.2 (1.44)	1.04 (1.62)	2.05 (1.37)	− 0.15	2.46 (1.66)	1.62 (0.87)	2.10 (1.46)	− 0.36
	2.0 (2)	1.5 (3)	1.0 (2)	− 1.0	2.0 (3)	1.0 (2)	1.0 (2)	− 1.0
EQ5D usual activities	100%	100%	86.4%		97.8%	61.9%	87%.	
	2.64 (1.45)	3.0 (1.70)	2.54 (1.45)	− 0.1	2.31 (1.31)	2.08 (1.12)	2.35 (1.44)	0.04
	2.0 (3)	3.0 (4)	2.0 (3)	0	2.0 (2)	2.0 (2)	2.0 (2)	0
EQ5D pain/discomfort	100%	100%	86.4%		100%	61.9%	89.1%	
	2.14 (1.07)	1.80 (1.03)	1.95 (1.01)	− 0.19	1.96 (0.89)	1.38 (0.77)	1.90 (0.94)	− 0.06
	2.0 (2)	1.5 (1)	2.0 (2)	0	2.0 (2)	1.0 (1)	2.0 (2)	0
EQ5D anxiety/depression	100%	100%	86.4%		97.8%	61.9%	89.1%	
	1.3 (0.63)	1.3 (0.48)	1.50 (0.76)	0.2	1.57 (0.86)	1.31 (0.48)	1.41 (0.81)	− 0.16
	1.0 (0)	1.0 (1)	1.0 (1)	0	1.0 (1)	1.0 (1)	1.0 (1)	0
EQ5D utility score	100%	100%	84.1%		95.7%	61.9%	87%	
	0.664 (0.234)	0.649 (0.204)	0.695 (0.227)	0.031	0.673 (0.262)	0.808 (0.186)	0.731 (0.246)	0.058
EQ5D health state	84.1%	60%	65.9%		87%	52.4%	65.2%	
	62.62 (21.45)	63.33 (12.52)	68.66 (17.01)	6.04	67.58 (23.35)	71.45 (22.05)	69.77 (22.03)	2.19
EQ5D health state box	90.9%	90%	81.8%		91.3%	61.9%	82.6%	
	62.63 (21.56)	64.44 (12.36)	71.53 (20.18)	8.9	67.50 (23.12)	73.08 (21.07)	70.13 (21.2)	2.63
ICECAP	100%	100%	100%		86.4%	61.9%	89.1%	
	0.737 (0.419)	0.6359 (0.2514)	0.811 (0.285)	0.074	0.767 (0.310)	0.7107 (0.2395)	0.788 (0.309)	0.021
FES	100%	100%	86.4%		100%	61.9%	89.1%	
	14.25 (6.16)	14.7 (5.27)	14.24 (6.31)		12.98 (5.44)	14.08 (6.78)	13.29 (6.52)	
	13 (8.5)	14 (10.5)	14 (10.25)	1	11(6)	11 (11)	10 (10)	− 1.0
CWIS physical symptoms and daily living/120	N/a	100%	50%		N/a	61.9%	70%	
	N/a	28.7 (14.85)	26.25 (4.5)	− 2.45	N/a	26.62 (9.2)	32.0 (8.35)	5.38
	N/a	24.5 (18)	24 (6.8)	− 0.5	N/a	28 (12.5)	30 (4)	2.0
CWIS social life/70	N/a	100%	50%		N/a	61.9%	70%	
	N/a	15.8 (13.59)	15.0 (2.0)	− 0.8	N/a	15.54 (5.75)	18.0 (6.51)	2.46
	N/a	7.5 (18.75)	14 (3)	6.5	N/a	15 (4.5)	15 (6)	0
CWIS wellbeing/35 (high score is worse)	N/a	90%	75%		N/a	61.9%	70%	
	N/a	11.26 (4.73)	10.67 (4.32)	− 0.59	N/a	15.84 (4.45)	17.14 (5.15)	1.3
	N/a	15 (7)	9.5 (7.25)	− 5.5	N/a	17 (8)	18 (5)	1.0
CWIS quality of life/20 (high score is better)	N/a	100%	62.5%		N/a	61.9%	70%	
	N/a	13.5 (3.95)	11.8 (4.03)	− 1.7	N/a	15.15 (3.21)	12.43 (6.19)	− 2.72
	N/a	14.5 (3.5)	14 (8)	− 0.5	N/a	16 (5.5)	14 (13)	− 2.0

### Contamination

Any future trial needs to consider the possibility of contamination, which we assessed in this pilot. The socks are commercially available on the internet and the study Participant Information Sheet would have drawn attention to their existence for participants, their relatives/carers and potentially their GPs. Any contamination that occurred in this pilot was recorded, and there was only one case of a patient in the TAU group obtaining a pair of socks for himself; he had previously received 8 repeated skin tears in a 2-month period.

## Discussion

This pilot addressed the uncertainties in planning a future definitive randomised controlled trial. The areas of uncertainty to be addressed are typical in a study of this nature: the feasibility of recruitment (of care homes, general practices and of individual participants), the suitability of outcome measure assessments and their timing and the distribution of variables (and therefore the number of participants needed in a full substantive trial of effectiveness). In particular, it focussed on the acceptability of the socks. Preliminary work and early patient and public involvement indicated that they might not be aesthetically pleasing to participants because of their current limited colour range (charcoal grey or beige) and perceived thickness. Doubts had also been raised about comfort and fitting as the size is currently limited to four (small, medium, medium-wide and large). We also did not know whether the socks can be worn and tolerated during the different seasons of the year.

Working in geographical cohorts proved to be efficient for research nurses but recruitment from care homes was slow due to mental capacity issues among residents and some reluctance to take part in research. Once recruited, however, residents enjoyed the weekly contact with the research nurses by phone and in person. In order to reach our target sample, participants were recruited from the community (GP practices) but they were on average 10 years younger than those from care homes (median 80 versus 90 years respectively). However, it was easier to recruit participants for the trial from the community because consenting one person only required two people to be invited. In care homes, it required six residents to be invited. Running clinics in GP surgeries was also less costly on travel and research nurse time where they could see five patients in a single morning. Seventy-nine participants (88%) completed the trial and 27/44 (61%) in the socks group wore the socks for the full 112 days. Of the two colours of socks available, the preferred colour was beige, although a larger proportion of men than women preferred charcoal grey. There were 11 withdrawals from the trial: 6 (14%) participants in the socks group and 5 (11%) in the control group. Of the questionnaire booklets, 100% were completed at baseline and 88% at 16 weeks. The best completed measure as a whole was the EQ5D5L.

There were 31 skin tear injuries affecting 18 of the original 90 participants (20%, 95% CI 12.3 to 29.8%) during a 16-week period. The incidence of skin tears was lower in care homes than in the community dwellers: RR 1.33 (0.73 to 2.18). In interviews, the latter confirmed that they were more active and ventured outside more than participants living in care homes. They were likely to be engaged in outdoor activities such as gardening and their injuries often related to these activities. Research nurses reported that the socks made the wearers feel protected and less cautious about attempting these activities. There were 21 skin tear injuries among 10 participants in the usual care group and 10 tears among 8 people in the socks group. The usual care group received more tear injuries, more repeated episodes, larger tears and more severe tears which would require emergency treatment. Only 2 of the 8 people in the socks group who received skin tear injuries were wearing their intervention socks when the injuries occurred. Furthermore, one of them was a care home resident whom carers stated habitually rolled down their protective socks, especially during the hot weather. Twelve adverse events had a causal relationship to wearing the socks: 8 were mild (mostly lower leg discomfort) and 4 were moderate (pain, swelling and a blister) but all recovered.

Daily diary reasons for not wearing the socks were varied but a large proportion of comments referred to the weather being too hot to wear them. However, these comments were made by only 4 of 44 socks group participants (9.1%) who were all recruited during the record-breaking hot summer of 2014 [47]. The qualitative interviews corroborated what participants had written in their daily diaries—especially concerning the perceived warmth of the socks. With regard to assessment of skin tear severity, the STAR severity grading system [43] fared well against the Payne-Martin system [1], achieving ‘very good’ agreement by research nurses.

Strengths of this pilot trial were that it recruited participants to the target sample within the time and budget available. It included residents of care homes, which represent an under-researched population of older people, but such recruitment was difficult and much easier in the primary care community. Dementia was an exclusion criterion in this pilot, which limited recruitment from care homes; moreover, older people with dementia are at even greater risk of skin tears than those without it [8]. A future trial should consider how best to include this group.

Randomisation using smart phones connecting to the CTU server was very effective and efficient. Furthermore, retention and concordance with the preventative measure were reasonable. Data on the primary outcome was able to be captured successfully, and completeness of questionnaire-based outcomes was also high. Diaries were less well completed.

Within this pilot the scope for contamination was seen to be low, but still a consideration for a future larger trial. Cluster randomisation would not prevent control participants seeking to purchase their own socks, but might reduce the potential for such contamination, e.g. because of seeing intervention patients wearing socks. However, cluster randomisation has disadvantages, and on balance, we recommend that a future trial uses individual randomisation.

No known studies of prevention of skin tears to the legs using socks woven with Kevlar exist. Current attempts at prevention are not very effective. As skin tears are caused by blunt trauma and shearing or friction forces on the skin, methods of preventing them concentrate on ways of avoiding these traumas to the skin and include [49–53]:

- Reviewing the hazards present within the individual’s environment and carrying out a risk assessment in order to minimise risk
- Padding hard surfaces, including wheelchair legs and any devices used to move the individual and dressing them in trousers, long-sleeved tops, knee-length socks and keeping all finger and toenails short and filed
- Educating the people responsible for caring for the individual in methods of gentle manual handling whilst turning and transferring and using lift sheets to move them in bed
- Reviewing medication to avoid polypharmacy, which has been identified as a risk factor for falls.
- Using a hypoallergenic moisturiser to maintain hydration for maintenance of skin integrity

## Assessment of severity

There is little consistency in how skin tears are assessed and documented. Le Blanc et al. [49] found that many of the respondents did not use a tool or system for classifying or documenting a skin tear. This could also lead to an under-reporting of skin tears and so the prevalence could, in fact, be much higher than previously thought.

Although the Payne-Martin classification system [1] has been validated both internally and externally, the results were not published (K. LeBlanc, pers. comm) and this may be why it has not been widely used in clinical practice [54, 55]. In a large-scale international survey of healthcare professionals’ practice and management of skin tears, only 10% of those surveyed said that they used the classification system in practice [54]. In fact, in our pilot study, the STAR grading system [43] provided similar inter-rater agreement to Payne-Martin [1] but better intra-rater agreement. The STAR grading system also identified more grade 2b and fewer 2a tears than the Payne-Martin.

## Risk factors

A recent systematic review described the risk factors for skin tears, which were most commonly a history of skin tears, impaired mobility and impaired cognition. Skin characteristics associated with skin tears included senile purpura, ecchymosis and oedema [16]. The group of individuals most at risk of developing a skin tear are those with impaired mobility who rely on others for help with activities of daily living (ADL), such as dressing, bathing and transferring from one environment to another [55]. Also at high risk are individuals who have impaired cognition and vision.

Following this, elderly individuals living independently are also at a high risk through falls, trips and trauma injuries through bumping into household and garden furniture [36]. The list of causes of skin tears witnessed in this pilot study includes all of these. There was a noticeable difference between care home residents whose injuries were more related to bedtime transfers and night-time activities and the younger, more active community participants, whose injuries were more related to activities outside (e.g. in the garden).

We selected community dwellers to approach for this study based on searching on general practice patients who had a history of long-term corticosteroid use. There was an evidence base for this. Long-term use of corticosteroids is thought to cause changes to collagen synthesis, thus increasing the susceptibility of the skin to tearing [54–57]. Corticosteroids are thought to affect various components of the extracellular matrix (ECM) which consists mostly of type I collagen fibres, and thus prevent the dynamic remodelling of skin and this contributes to skin atrophy [58] and also reduce the synthesis of epidermal lipids, thus increasing transepidermal water loss.

Topical use of corticosteroids can lead to skin atrophy, characterised by a decrease in skin thickness and elasticity, telangiectasia and purpura [59]. Glucocorticoids reduce proliferation of keratinocytes and have an adverse effect on their size [60–62]. This is accompanied by a decrease in the proliferation of fibroblasts and also production of ECM proteins [63–66].

Use of anticoagulants has been linked to changes in the skin such as haematomas, senile purpura and ecchymoses [1, 54, 55]. It has previously been observed that skin tears occur often at the site of senile purpura [51, 65, 66], and ecchymosis has been identified as an extrinsic independent risk factor for skin tears [52]. Haematomas compromise the viability of adjacent tissue, and this can be exacerbated by the use of warfarin [67].

Skin tears often occur at sites of previous ones. This is likely due to the healed site having a reduced tensile strength [49, 68]. Other conditions associated with an increased risk of developing skin tears include oedema and diabetes mellitus. This is thought to be due to the final common pathway of oxidative stress and over-expression of MMPs, matrix metalloproteinase enzymes that play an important part in wound healing [36, 69]. Individuals with diabetes mellitus are also more likely to be at risk of delays in wound healing.

## Implications for this study

The protective socks offer hope of some protection from skin tears when very few other measures have worked and this pilot indicates that it is worth carrying out a full-scale definitive trial in the future to determine their effectiveness and cost effectiveness.

Individuals at highest risk of developing skin tears are those who are dependent on others for their activities of daily living, such as dressing, bathing, transferring and re-positioning. Many of these individuals are residents in care homes, but some will also be found in the community. Another group at high risk of skin tears are those ambulatory individuals who either are taking medications (e.g. long-term use of steroids or anticoagulants, or have chronic conditions (e.g. cardiovascular or respiratory disease) which render their skin more susceptible to skin tears. The participants in the next trial would, therefore, include these groups of eligible individuals.

For a future trial, we would consider asking the participants to wear the socks at night time as well as the day. In this pilot trial, injuries sustained by people in the socks group mostly occurred when the person was not wearing them, for example at night when a person may get out of bed and blunder into an unseen obstacle.

We would also encourage the manufacturer of the socks to provide a larger range of colours of socks in a wider range of sizes as limited colour choice appeared to be a barrier to participation and the available sizes did not fit a few people. Also, there was a perceived need for thinner socks to cater for hot summer weather. However, changing the thickness may affect any protective properties that the socks appear to have.

The feasibility data recorded in this trial will inform the design of a further definitive trial to investigate whether protective leg (and possibly arm) wear can prevent skin tears in high risk individuals. We intend to apply for funding from NIHR or another major funding body.

## Implications for future research

The most appropriate primary outcome measure for the future RCT are not skin tear-free days but the incidence of skin tears including their size and severity (as scored by the Payne Martin and/or STAR rating system), together with the EQ5D5L and Cardiff Wound Inventory as a secondary outcome measures.

This pilot trial demonstrated an effect of 8% points between groups (effect size 0.40) even although only 61% of participants achieved 100% compliance with wearing of the socks and also not at night. Given the pragmatic nature of this trial, in a future trial we would introduce mechanisms (encouragement phone calls) to further encourage adherence with trial procedures.

Refinements to the intervention arising from qualitative work on acceptability could include the availability of a greater range of sock colours, which might help to improve recruitment.

Regarding cost and effectiveness measures, it was not difficult to obtain the cost of dressings to treat skin tears seen by community nurses. It was more difficult to obtain accurate measurements of amounts of time spent by various professionals in treating the wounds.

## Sample size for a future study

A future study would need to be multi-centre to recruit the required numbers of participants. It would need to be conducted in primary care because recruitment is easier than in care homes and also this community population are more active and exposed to more skin tear hazards in their daily lives. Based on the difference in incidence found in this pilot, for 90% power, this would require 880 patient recruits.

## Conclusions

We have shown that it is possible to recruit and retain sufficient participants to conduct a trial of skin tear prevention using novel protective socks in care homes and the community.

The results from this trial are encouraging, and we plan to conduct a future trial to investigate whether these protective socks/leggings are effective and cost-effective in protecting against skin tears to the lower legs.
